# Understanding Heterogeneity in Clinical Cohorts Using Normative Models: Beyond Case-Control Studies

**DOI:** 10.1016/j.biopsych.2015.12.023

**Published:** 2016-10-01

**Authors:** Andre F. Marquand, Iead Rezek, Jan Buitelaar, Christian F. Beckmann

**Affiliations:** a Donders Centre for Cognitive Neuroimaging, Donders Institute for Brain, Cognition and Behaviour, Radboud University, Nijmegen, The Netherlands; b Department of Cognitive Neuroscience, Radboud University Medical Centre, Nijmegen, The Netherlands; c Department of Neuroimaging, Centre for Neuroimaging Sciences, Institute of Psychiatry, King’s College London, London, United Kingdom; d Karakter Child and Adolescent Psychiatric University Centre, Nijmegen, The Netherlands; e Oxford Centre for Functional Magnetic Resonance Imaging of the Brain, University of Oxford, OxfordUnited Kingdom; f Schlumberger Gould Research Center, Cambridge, United Kingdom

**Keywords:** Gaussian process, Heterogeneity, Normative model, Outlier detection, Patient stratification, Research Domain Criteria

## Abstract

**Background:**

Despite many successes, the case-control approach is problematic in biomedical science. It introduces an artificial symmetry whereby all clinical groups (e.g., patients and control subjects) are assumed to be well defined, when biologically they are often highly heterogeneous. By definition, it also precludes inference over the validity of the diagnostic labels. In response, the National Institute of Mental Health Research Domain Criteria proposes to map relationships between symptom dimensions and broad behavioral and biological domains, cutting across diagnostic categories. However, to date, Research Domain Criteria have prompted few methods to meaningfully stratify clinical cohorts.

**Methods:**

We introduce normative modeling for parsing heterogeneity in clinical cohorts, while allowing predictions at an individual subject level. This approach aims to map variation within the cohort and is distinct from, and complementary to, existing approaches that address heterogeneity by employing clustering techniques to fractionate cohorts. To demonstrate this approach, we mapped the relationship between trait impulsivity and reward-related brain activity in a large healthy cohort (*N* = 491).

**Results:**

We identify participants who are outliers within this distribution and show that the degree of deviation (outlier magnitude) relates to specific attention-deficit/hyperactivity disorder symptoms (hyperactivity, but not inattention) on the basis of individualized patterns of abnormality.

**Conclusions:**

Normative modeling provides a natural framework to study disorders at the individual participant level without dichotomizing the cohort. Instead, disease can be considered as an extreme of the normal range or as—possibly idiosyncratic—deviation from normal functioning. It also enables inferences over the degree to which behavioral variables, including diagnostic labels, map onto biology.

The case-control approach to studying brain disorders has been successful for detecting group effects, for example, between patients and control subjects. However, it becomes problematic in domains such as psychiatry where disorders are diagnosed on the basis of symptoms that overlap between disorders, often yielding clinical groups that are heterogeneous and overlapping. This problem is particularly acute in psychiatry because biological tests to assist diagnosis or predict outcome have not been developed (1). Moreover, the case-control paradigm induces an artificial symmetry such that both cases and controls are assumed to be well-defined entities (Figure 1). This does not match the clinical view of disease, where disorders in individual patients manifest as deviations from a normal pattern of functioning.

In response to this problem, the National Institute of Mental Health launched the Research Domain Criteria (RDoC) initiative (2), which encourages researchers to link symptom dimensions with biological systems, cutting across diagnostic classifications. The ultimate aim of RDoC is to find “new ways of classifying psychiatric diseases based on multiple dimensions of biology and behavior” (http://www.nimh.nih.gov/research-priorities/rdoc/index.shtml)—reducing heterogeneity in clinical cohorts; improving the neurobiological validity of disease classifications; and enabling more effective, personalized treatments. These objectives are also consistent with the European roadmap for mental health research (3). These objectives are difficult to achieve within the case-control paradigm, which, by definition, entails partitioning cohorts according to predefined labels, precluding later inferences about their validity.

The RDoC initiative has prompted considerable discussion (4, 5) but to date has led to few methods to study heterogeneity within clinical cohorts. Of the reports published, nearly all have employed data-driven clustering methods aiming to fractionate clinical groups mostly on the basis of neuropsychological measures. For example, clustering methods have been applied to subtype attention-deficit/hyperactivity disorder (ADHD) (6, 7, 8, 9), mood disorders (10, 11), and schizophrenia (12, 13). Clustering is useful for identifying subgroups of participants at a particular time point but also has problems: 1) there are many different ways to partition clinical populations depending on the measures and clustering algorithm used; 2) some participants may not clearly belong to any class, or some classes may become unmanageably small (8); 3) patient subgroups may not be stable over time (14); 4) it may be difficult to choose a unique optimal number of clusters (e.g., different metrics may yield different optimal numbers of clusters or may not identify a unique maximum); 5) finally, it is unclear whether healthy participants should be clustered separately or in combination with patients. Some reports have suggested that disease variation may be nested within normal variation (7).

In this article, we propose an alternative conceptual advance for parsing heterogeneity in clinical and healthy cohorts. In contrast to clustering approaches, we propose a normative modeling approach that models biological variation across either 1) the entire study population (including all clinical groups) or 2) a large healthy sample. The intuition is that by mapping the full range of population variation, we can consider symptoms in individual patients as an extreme value within this distribution. This is analogous to the use of growth charts to map child development in terms of height and weight as a function of age, where deviations from a normal growth trajectory manifest as outliers within the normative range at each age. This approach is fundamentally different from, and complementary to, clustering (Figure 1). More concretely, we predict biological measures of brain function (e.g., neuroimaging) on the basis of clinically relevant covariates (e.g., trait measures). We build on preliminary work by ourselves and others (15, 16, 17, 18) to introduce an analytical framework that allows us to 1) use data from large cohorts to learn a normative distribution that characterizes the study population; 2) make probabilistic statements about which participants deviate from the normative pattern; and 3) statistically map the brain regions underlying these deviations on a case-by-case basis, while permitting 4) diagnostic labels to be used as predictor variables, enabling inferences over the labels just as any other variable.

To illustrate, we map the relationship between trait impulsivity and reward-related brain activity in a large, healthy sample. This relationship is of high clinical relevance because impulsivity and impairments in reward processing are core features of many disorders, including ADHD (19, 20) and addiction (21). We use delay discounting to quantify impulsivity, which measures the degree to which individuals devalue future rewards relative to immediate rewards (22) and is a stable measure of trait impulsivity (23). We then relate the model predictions to ADHD symptom dimensions to highlight specificity for particular symptom domains. Our approach is predicated on the assertions that 1) understanding healthy variation is a prerequisite to understanding disease variation and that this requires 2) the ability to determine where each subject lies within the population range because variation associated with most disorders overlaps with normal variation. We show that normative modeling provides a flexible and powerful means to operationalize these desiderata, to study variation in individual participants, and to highlight axes of variation relevant to clinical symptoms.

## Methods and Materials

### Overview of Normative Modeling

Figure 2 shows an overview of the approach. First, we estimate a normative model that links clinical and biological variables. Specifically, we use Gaussian process regression (24) to predict a set of biological response variables (e.g., neuroimaging) from a set of clinically relevant covariates (e.g., trait scores), while estimating predictive confidence for every prediction. Measures of predictive confidence are important because they quantify the fit of each point to the normative model in that centiles of predictive confidence can be interpreted as centiles within the normal range (Figure 2A). A normative (expected) trajectory is provided along with expected modes of variation, linking the trait variable to the biological response variable. This model can provide predictions for any value of the clinical covariates, whether observed or not, and by evaluating the entire range of all covariates, we can derive disease spectra that describe the full range of normal variation (17). Our focus in this study is on charting variation across clinical predictor variables, but related multivariate regression approaches have been used to predict subject age with respect to a normal developmental trajectory (18, 25, 26, 27, 28).

### Estimating the Normative Model

Gaussian process regression is described in Supplement 1, but briefly, a Gaussian process is a distribution over functions that can be used for Bayesian interpolation. In this study, we model the relationship between delay discounting and reward-related brain activity independently for each brain region (“brainordinate”).^1^ That is, we specify a functional relationship between a vector of covariates (**x**, here “delay discounting”) and responses (*y*, here “brain activity”):

y=f(x,θ)+∈

The residuals are denoted by ∈, and **θ** is a vector of parameters. The parameters control the scale of the function used to interpolate the data and the relevance of each covariate for predicting the response variable. Thus, irrelevant covariates can be down-weighted, and relevant covariates can be emphasized. We place a Gaussian process prior over the set of interpolating functions and compute their posterior distribution by Bayes’ rule. This approach provides three advantages: 1) it is highly flexible and can accommodate nonlinear relationships; 2) it provides coherent estimates of predictive uncertainty; and 3) it delivers state-of-the-art prediction performance in many domains, including neuroimaging (29, 30). This approach is applicable to most types of neuroimaging data (e.g., structural magnetic resonance imaging [MRI], functional MRI) both at the voxel/vertex level and using regional summary measures (e.g., average activity within anatomically defined masks or maximum cluster volume).

Next (Figure 2B), we use the normative distribution to make a prediction for each subject, either from a new cohort or from the same cohort but excluded from the model (e.g., under cross-validation). For each subject (*i*), the prediction at each brain location (*j*) consists of an expected response (mean, ${\hat{y}}_{ij}\;$) and expected level of variation (variance, $\sigma_{ij}$). This can be combined with the true response ($y_{ij}$) and variance learned from the normative distribution ($\sigma_{nj}$) to provide a *Z* score quantifying the deviation from the normative model:

$$z_{ij}=\frac{y_{ij}-{\hat{y}}_{ij}}{\sqrt{\sigma_{ij}^{2}+\sigma_{nj}^{2}}}$$

This is known as a normative probability map (NPM) e., what does "This" refer to?) -->(16) and combines three sources of information: 1) the error (difference between true and predicted responses); 2) the predictive variance of the test point; and 3) the variance of the normative data. The model accommodates the distribution of the data such that predictions made in regions of the input space with a low density or high spread of points will have appropriately low predictive precision (high variance). This feature is important in many clinical contexts; for example, it may be difficult to obtain a cohort that covers the complete range of the covariates of interest. Ideally, it is desirable to have a large sample with good coverage of the full range, but this may not be possible (e.g., because it may require recruiting many low-functioning patients). Our approach guarantees that uncertainty is handled coherently, regardless of the context (31). Thus, the model automatically reduces predictive confidence when extrapolating away from the data points or in regions where variability within the normative cohort is high. Finally, because the NPM *Z* scores are estimated independently for each brain region, conventional statistical machinery can be used to control the type I error rate.

### Summarizing Deviations Into a Subject-Level Abnormality Index

The above-described approach quantifies an individual participant’s response pattern, given their behavioral covariates. In the case of a multivariate response (e.g., having thousands of brain locations), this results in a multivariate measure of deviation. For subject-specific decision making, it is essential to summarize the degree of abnormality by estimating the total magnitude of deviation for each subject with respect to the normative model. To achieve this, we employ extreme value statistics, which model the behavior of random variables in the tail of their distribution; extreme value statistics have been applied, for example, to predicting unusually large floods or stock market crashes (32, 33). In this study, we consider that disease may—but may not necessarily—occur as an extreme deviation from a normal pattern. We adopt a “block maxima” approach (33) to modeling deviations that involves summarizing each block of data, or each participant, by his or her maximum value. To ensure that this approach is reliable, we compute a robust (90% trimmed) mean of the top 1% of NPM *Z* statistics, summarizing the deviation across all brain regions (Figure 2C). To make probabilistic subject-level inferences about these deviations, we fit an extreme value distribution (Supplement). It is also possible to consider signed deviations from the normative model, depending on whether the top 1%, bottom 1%, or top 1% absolute value of the distribution is taken (positive, negative, or absolute deviation). These deviations convey different information, and their interpretation in the context of an actual analysis depends on whether an underactivation or overactivation is expected to be related to clinical symptoms. In this study, we use the absolute deviation to quantify the total deviation from the normative model and signed deviations to examine correlations with clinical variables because to understand mechanisms underlying the deviation, it is necessary to differentiate cases having overactivation from cases having underactivation, relative to the normative model.

### Data Sample

Normative modeling aims to chart variation within the population distribution so that deviations can be reliably assessed. Therefore, we employ a large healthy sample for whom high-quality data are available. For this study, we use data from 491 participants (288 females; mean age 27 years [range 22–36 years]) from the Human Connectome Project (http://www.humanconnectome.org) (34, 35, 36). See the Supplement for details of the sample characteristics, task design, data acquisition, and processing. Briefly, all participants completed the following: 1) a functional MRI incentive processing (gambling) task (37); 2) the Achenbach Adult Self-Report instrument (38), used to measure clinical symptoms on the basis of DSM-IV criteria; and 3) a delay discounting task (39) that quantifies the extent to which future rewards are devalued relative to immediate rewards at two delayed reward magnitudes: $40,000 and $200. The area under the curve (AUC) (40) was used to summarize delay discounting across all delays evaluated for each magnitude (AUC40K, mean 0.46 [range 0–0.98], and AUC200, mean 0.24 [range 0–0.98]). The means for ADHD symptom scales were 3.03 (range 0–11) for inattention, 2.37 (range 0–14) for hyperactivity, and 5.40 (range 0–25) for total scores.

We use delay discounting to quantify trait impulsivity, which serves as a covariate to predict the biological response variables (functional MRI contrast images between reward and baseline). We then relate images to the Adult Self-Report scales for inattention and hyperactivity. To ensure unbiased estimates of generalizability, model estimation was performed under 10-fold cross-validation, where data were repeatedly partitioned into training and test sets across subjects, in a way that accommodated the family structure of the data (Supplement).

## Results

### Overview of Normative Model

Measures of delay discounting are presented in Figure 3. No delay discounting measures were correlated with either hyperactivity (AUC200, *r* = −.047; AUC40K, *r* = −.040, both *p* > .2) or inattention (AUC200, *r* = .079; AUC40K, *r* = .045, both *p* > .2). For illustrative purposes, the principal direction of variance in Figure 3 is indicated by an arrow. Several reference points along this direction (numerals) provide anchors for an exemplar spatial representation of the normative model (Figure 4), defined with respect to a theoretical baseline participant that does not discount reward at all (the point marked “B” in Figure 3).

The normative model predicts that participants who discount rewards more strongly also more strongly engage a network of cortical and subcortical regions (described in the Supplement). This network overlaps with, but is not identical to, the network activated by the gambling task [Figure 6 in Barch *et al.* (39); see also Figure 6A later on]. It is also consistent with the network engaged by delay discounting (41) and, as expected, corresponds to the regions that the normative model predicted accurately under cross-validation (Figure 4C).

### Clinical Correlates of Abnormality Index

Figure 5 shows the absolute deviance of each participant plotted against hyperactivity. This figure reveals structure in the data that is not apparent considering only symptoms (Supplemental Figure S1). First, most participants fit the normative model well, including many participants having high hyperactivity. To illustrate, we consider participants having hyperactivity >8 (Figure 5, blue circles). The normative model provided a good fit for these participants because none showed brainordinates deviating from the norm (*p* < .05, false discovery rate corrected), and none would be considered an outlier under the extreme value distributions fit to the positive, negative, or absolute deviations (all *p* > .05). Therefore, the normative model captures variation across the full range of hyperactivity symptoms.

Second, not all participants fit this pattern; other subjects have high hyperactivity but deviate from the normative model (Figure 5, red circles). These participants score in the 99th and 97th percentiles for hyperactivity within the cohort. Furthermore, a strong positive correlation (*r* = .91, *p* = .03) was found between the negative deviance and hyperactivity for the 1% of participants showing maximal deviation (*n* = 5). The degree of deviation was not only informative about the most extreme subjects because the correlation persisted into the bulk of the population, remaining significant for the participants having negative deviance in the top 5% (*r* = .53, *p* = .007, *n* = 25), 10% (*r* = .31, *p* = .03, *n* = 50), 15% (*r* = .36, *p* = .002, *n* = 74), and 20% (*r* = .22, *p* = .03, *n* = 99) of the cohort. None of the corresponding positive deviance scores correlated with hyperactivity (all *p* > .4).

In contrast, deviance was not associated with inattention. Subjects with the highest absolute deviance did not show high inattention (Supplemental Figure S2), and neither the positive nor negative deviance correlated with inattention (all *p* > .2). Therefore, normative modeling allows us to tease apart symptom domains that are highly correlated.

### Deviating Subjects

The NPMs for 22 participants contained brain regions deviating from the normative model (*p* < .05, false discovery rate corrected). To illustrate, the NPMs for the 10 most extreme outliers are shown (Figure 6 and Supplemental Figure S3). The patterns of abnormality were highly individualized with low overlap between subjects; no brain region deviated in more than three participants (Supplemental Figure S4), pointing toward significant heterogeneity of the imaging phenotype within this cohort. The extreme value distribution provides a mechanism to understand how these deviations relate to clinical symptoms. To illustrate, Supplemental Figure S5 shows one possible interpretation of the deviations plotted in Figure 5, where symptoms may arise through 1) mechanisms that are well captured by the normative model or 2) idiosyncratic deviations from the normative model.

## Discussion

In this article, we present a principled method to study associations between brain function and behavior. We used normative models constructed from spectra of clinically relevant variables (delay discounting) to predict reward-related brain activity in a large, healthy cohort. This approach allowed us to 1) map the range of normal variation in reward-related brain responses, 2) perform statistical inferences at the level of the individual participant, and 3) identify participants deviating from the normative model. We related the degree of deviance to ADHD symptoms and showed that participants scoring highly for hyperactivity were either at the extreme of a normal spectrum of functioning or had reward-related brain responses that differed from the normal pattern. In the latter case, the degree of deviation correlated with hyperactivity, but not inattention, well into the middle of the population distribution on the basis of idiosyncratic patterns of abnormality.

A key feature of our approach that differentiates it from other approaches to studying heterogeneity in clinical populations (7, 8) is the primary focus on mapping variation across the cohort. This mapping breaks the symmetry inherent in the case-control approach in that the primary interest is in how each individual differs from the population. There are four advantages to this mapping: 1) it allows differential effects from normal functioning to be studied in individual subjects, 2) it does not entail making strong assumptions about the clinical group (e.g., existence or number of subgroups), 3) it provides an intuitive match to the clinical conception of disease, and 4) it provides a principled bridge between “big data” analytics and “precision medicine” (42) in that large healthy cohorts can be used to progressively refine estimates of normal variation. In the present study, we aimed to examine variation within a single healthy cohort, quantifying where each individual lies within the population. Normative models are also useful for fractionating heterogeneous clinical groups, where the normative distribution can be fit to a large sample of healthy participants to learn a healthy normative pattern and then applied to a clinical cohort to determine where patients lie on the healthy continuum. Alternatively, the normative model can be fit directly to the heterogeneous cohort to find outliers within the cohort.

Normative modeling is compatible with the objectives of RDoC because it allows different axes of variation to be studied independently of the diagnostic labels. A particular strength is its flexibility; it can integrate multiple measures characterizing different cognitive domains, quantifying the relationship between each domain with biology and symptoms. This is of high clinical relevance for three reasons. First, many clinical categories are based on clinical algorithms, combining self-report with clinician and parent/teacher assessment. There is no consistent way to deal with these multiple measures other than to add them up. The abnormality indices we propose provide a way to assess this information quantitatively in relation to biology. Second, we can begin to make statements about the quality of different measures, which can be compared in terms of predictive power. Third, existing diagnostic labels can be included as covariates just as any other variable. This inclusion permits inference over the proportion of biological variance the labels explain and therefore how appropriate a case-control analysis is for the chosen measures. Normative models can also be targeted specifically to detect abnormalities in multivariate phenotypes by making different choices for variables used for the covariates or responses. For example, measures derived from brain regions or networks can be employed if prior information about disease pathology is available. In the absence of such information in the present study, we employed a spatially unbiased, whole-brain approach. Alternatively, Gaussian process regression can be extended to directly predict multivariate phenotypes, as we demonstrated previously (43, 44).

Normative modeling provides the ability to make statistical inferences at the level of the individual. It shares similarities with pattern recognition methods commonly used for predicting clinical variables from neuroimaging data (45). Most of these applications have employed supervised learning, which requires the diagnostic labels to be specified in advance. Although unsupervised methods do not require the labels to be specified in advance, they do entail making assumptions (e.g., orthogonality, independence, or similarity by some measure) that lead to different ways to partition groups. Therefore, it can be difficult to constrain unsupervised methods to identify clinically relevant variation instead of nuisance variation, especially in high dimensions. Normative modeling provides an appealing middle ground. Moreover, our approach has advantages over existing approaches to normative modeling based on multivariate regression (18) and the one-class support vector machine (15). Most importantly, our approach provides probabilistic predictions and the ability to make statistical inferences about the manner in which individual subjects differ from the normative model.

**Figure 1:**
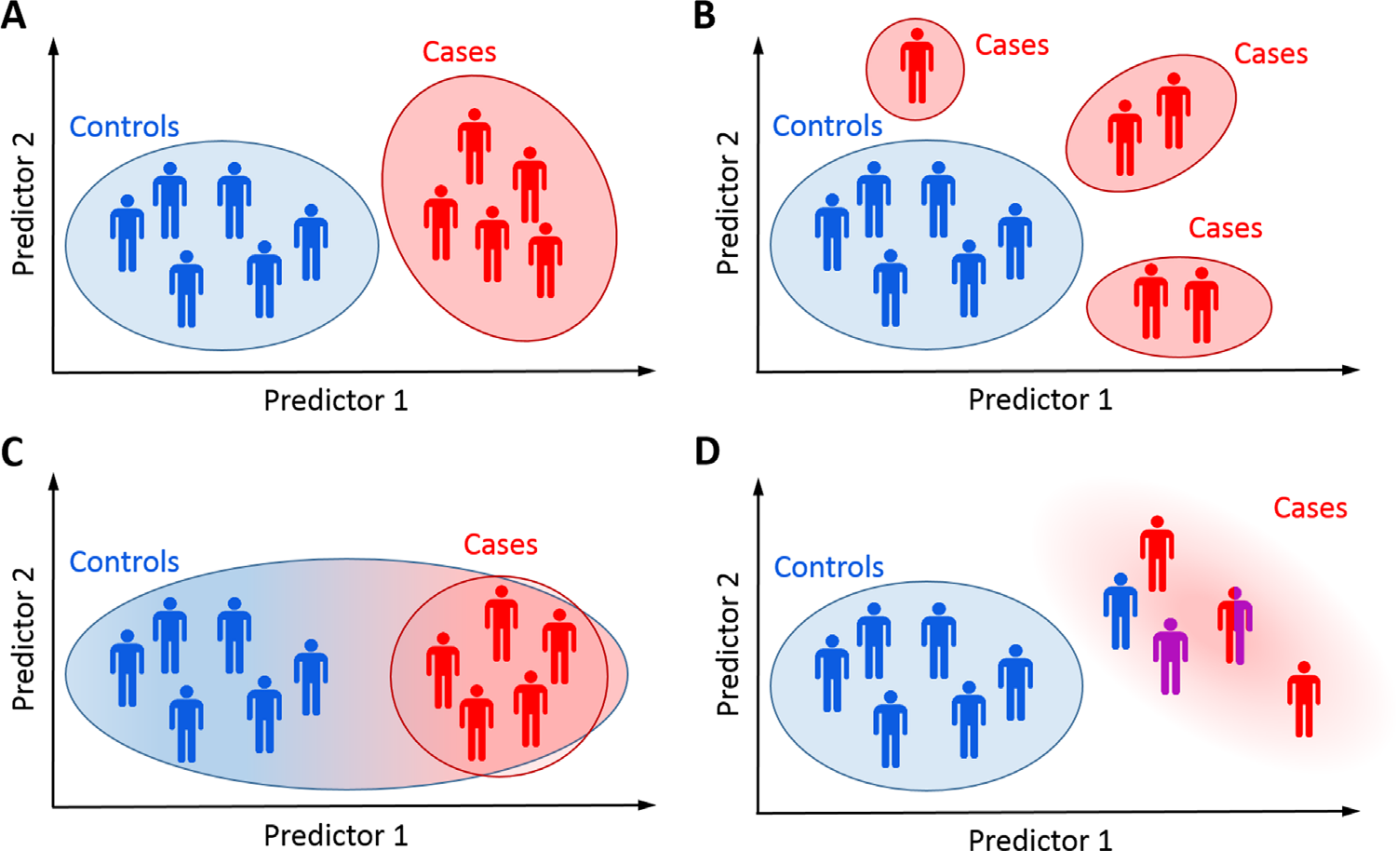
The classical case-control approach assumes that cases and controls each form a well-defined group **(A)**. This may often be a reasonable assumption, but in practice many other scenarios are possible. The clinical population may be composed of multiple groups, each having distinct pathology **(B)**; disease-related variation may be nested within healthy variation **(C)**; or the clinical group may be diffuse and heterogeneous as a result of misdiagnosis, comorbidities, or an aggregation of different pathologies **(D)**.

**Figure 2:**
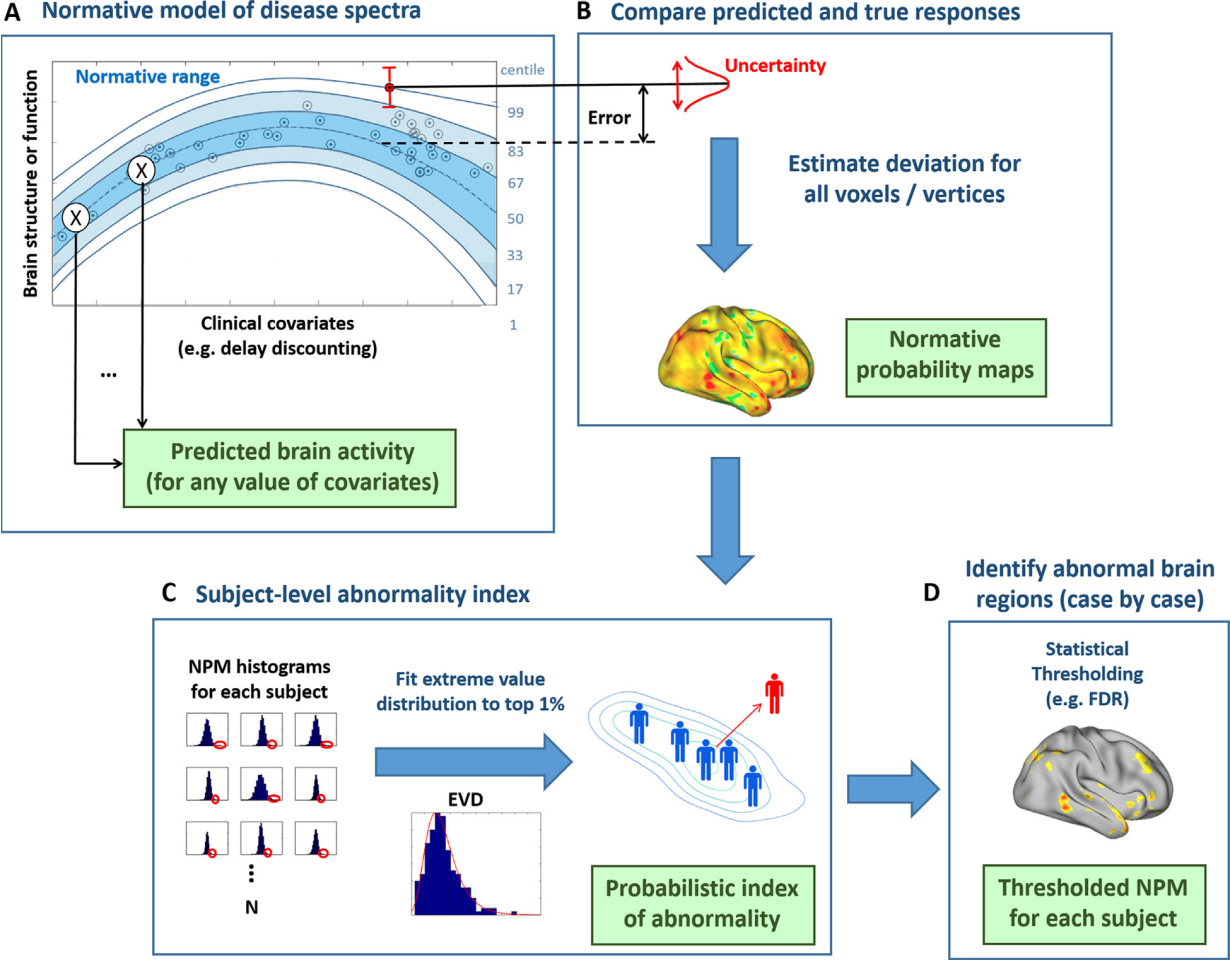
Overview of the proposed normative modeling approach showing the steps in the pipeline. **(A)** Estimate the normative model with Gaussian processes. This provides the ability to predict brain activity for any (observed or unobserved) value of the clinical covariates along with measures of predictive confidence (blue contour lines). The contours of predictive confidence can be interpreted as centiles of predictive confidence for the cohort (blue numerals, right). **(B)** For each subject, compute a normative probability map that quantifies the deviation from the normative model at each brain region. **(C)** Generate a summary measure of abnormality for each subject using extreme value statistics, which can be related to clinically relevant variables. **(D)** The imaging phenotype can be examined more closely, for example, by thresholding the normative probability maps using established techniques. This can provide insight into the brain mechanisms for subjects that do not fit the normative model. See text for full details. EVD, extreme value distribution; FDR, false discovery rate; NPM, normative probability map.

**Figure 3:**
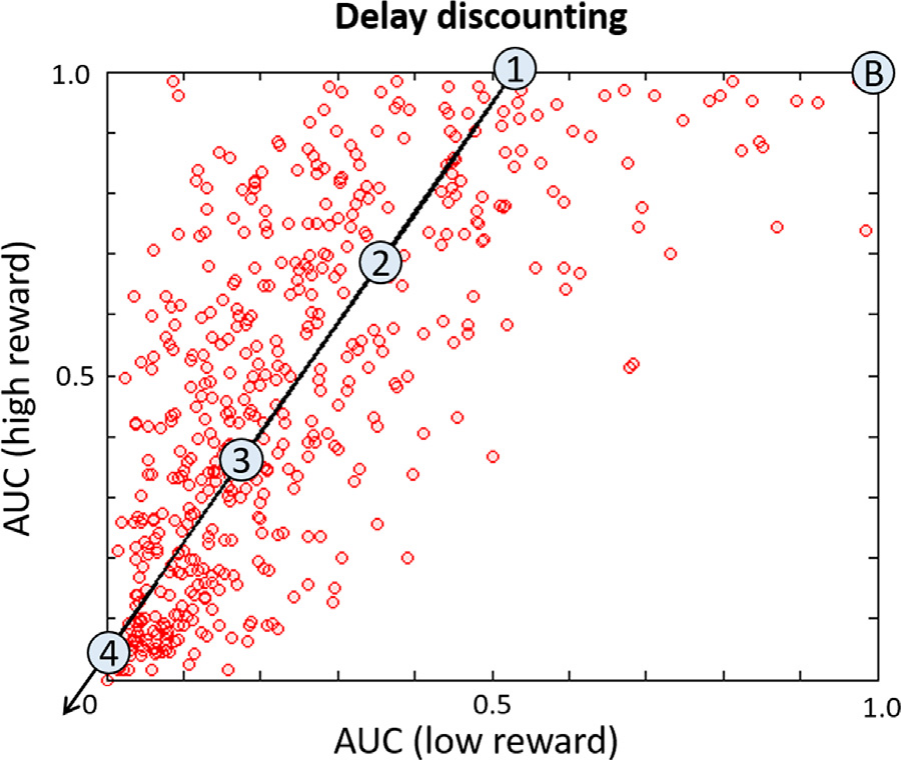
Area under the delay discounting curve (AUC) for the participants included in the normative model for low ($200) and high ($40,000) reward. For both reward levels, lower values are associated with steeper discounting of future reward. Participants discounted small rewards more than large rewards (*t*_491_ = −24.97, *p* < .001), and many participants strongly discounted both large and small rewards, depicted by a skew of the point cloud toward the y axis and an increasing density of points toward the bottom left corner, respectively. The arrow indicates an increase in overall delay discounting along the axis of maximum variance (i.e., principal eigenvector). The numbered circles indicate the positions selected for the spatial representation of the normative model relative to the baseline model labeled “B” (see text for details).

**Figure 4:**
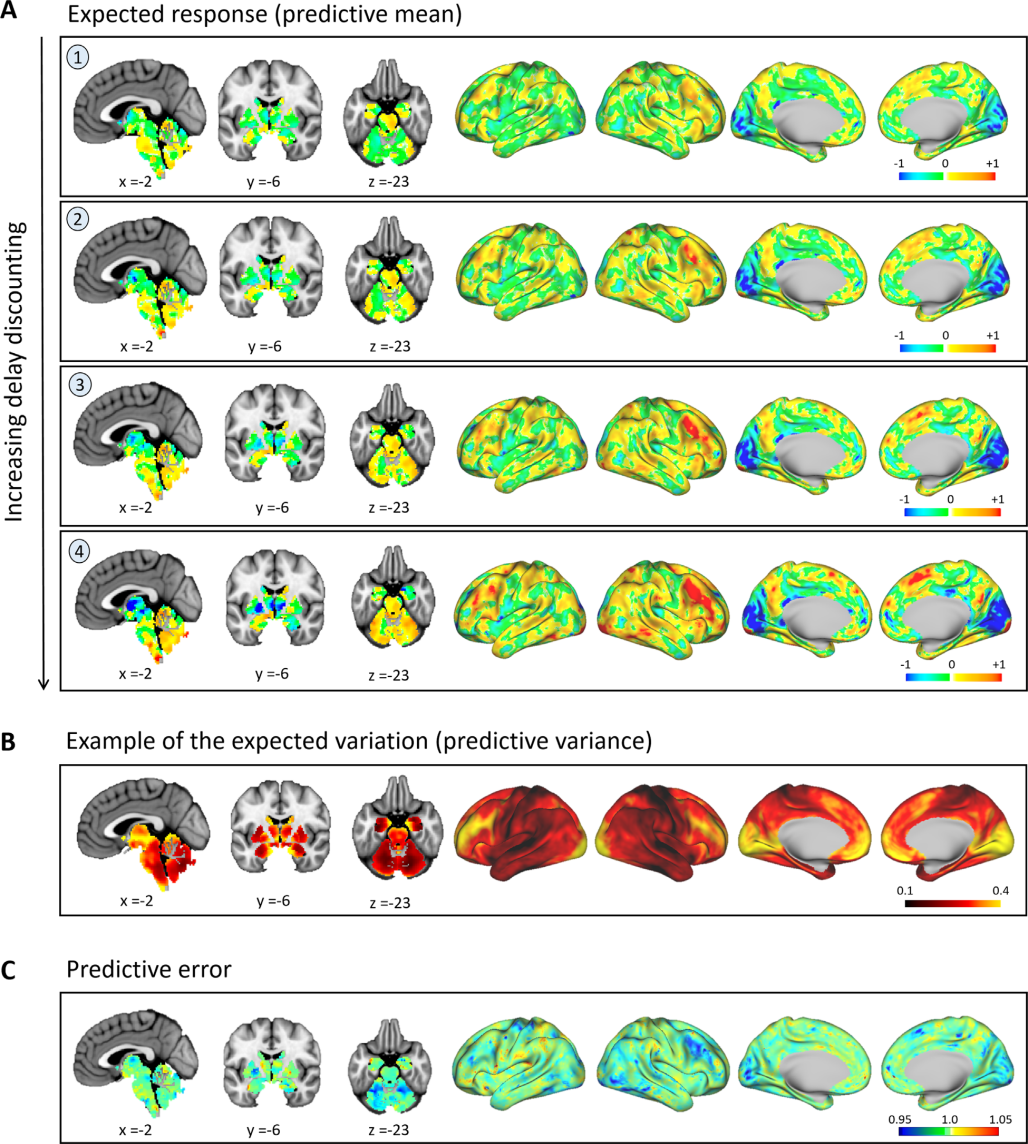
Spatial representation of the normative model. These maps show the predictions made by the normative model for the (fictitous) data points described in Figure 3, obtained after retraining the model using all available data. **(A)** The expected response. This shows increasing engagement of a network of brain regions with increasing overall delay discounting (rows). The numeral indexing in each row corresponds to the points in covariate space described in Figure 3. To assist visualization, these images have been rescaled such that the maximum across all images is equal to one. **(B)** An example of the expected variation, which was relatively constant for these points. This image has been rescaled such that the maximum variance in the image is equal to 1. **(C)** The standardized mean squared error for the normative model under cross-validation (averaged across all cross-validation folds). Comparison with **(A)** shows that the regions that could be accurately predicted (cool colors) correspond to regions that exhibit variation under different degrees of delay discounting.

**Figure 5:**
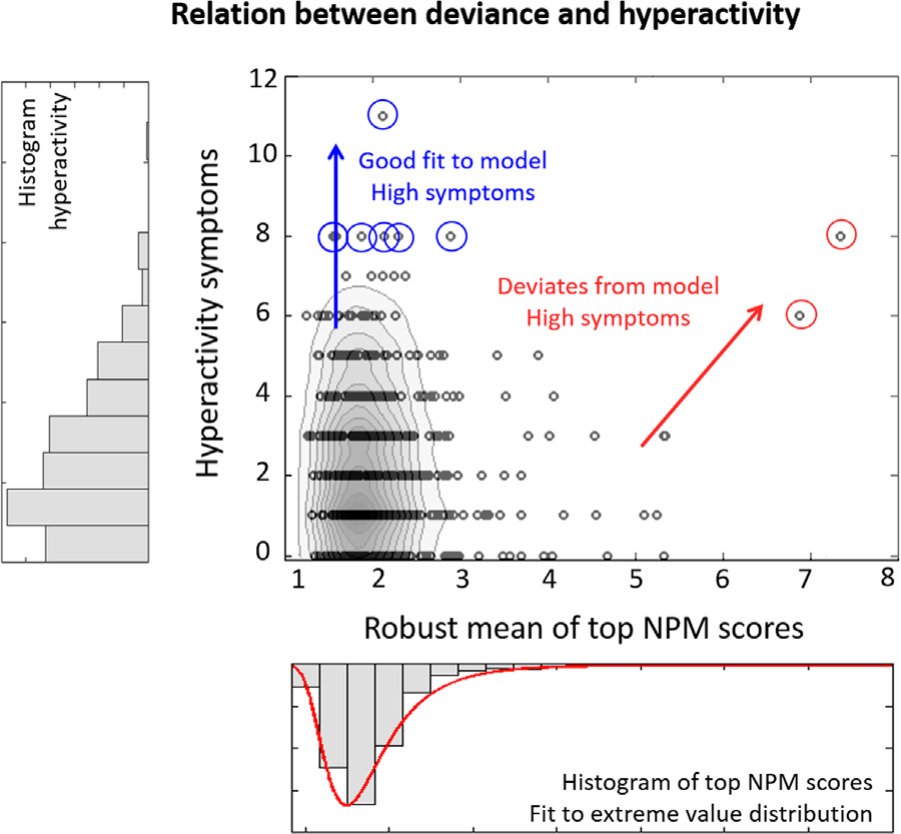
The relationship between the overall deviance from the normative model and hyperactivity scores (center), along with the histograms of the component measures (left and bottom). This figure allows us to determine whether subjects that have high clinical symptoms show a good or a poor fit to the normative model. For illustrative purposes, contour lines show the density of points in the figure. Most points fit the normative model well, but some of these subjects also score highly on hyperactivity (blue arrow). Other subjects who score highly on hyperactivity do not fit the normative model (red arrow). Circled subjects are discussed further in the text. NPM, normative probability map.

**Figure 6:**
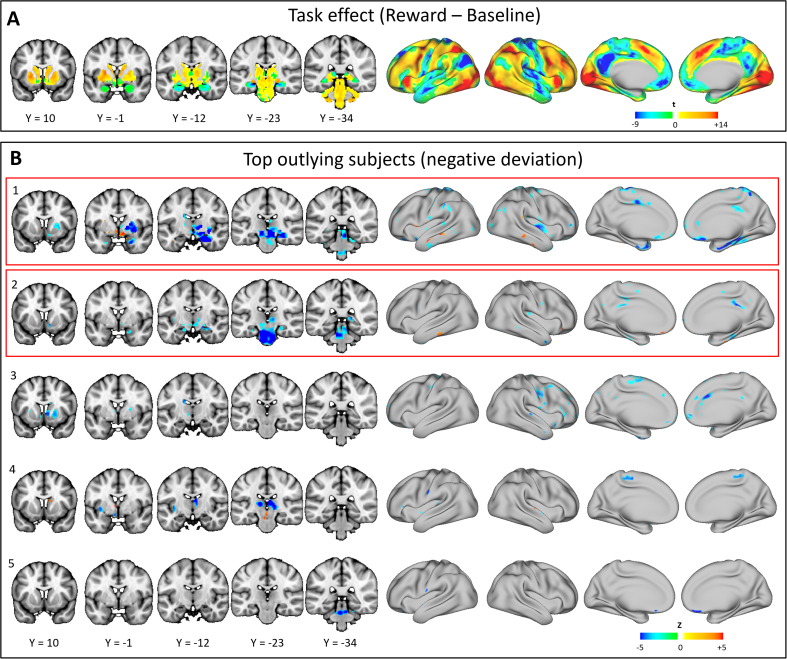
**(A)** An unthresholded *t*-statistic image of the main task effect, estimated using a classical general linear model (reward-baseline). Warm colors indicate greater activation during reward, and blue colors indicate reduced activation during reward. We have shown an unthresholded map because this sample has very high power (being estimated from nearly 500 subjects). Thus, nearly all brain regions survive conventional statistical thresholding. **(B)** Normative probability maps that describe the brain regions that deviate from the normative model in the 10 subjects having the most extreme deviations (*p* < .05, false discovery rate corrected) (also see Figure S3 in Supplement 1). Warm colors indicate greater activity than would be predicted by the normative model, and cool colors indicate reduced activity relative to the normative model. Subjects are ranked by hyperactivity symptom scores with the rank indicated by the numerals (1 = highest hyperactivity). The two most extreme deviations circled in Figure 5 are indicated by red boxes.

Normative modeling complements clustering approaches; it can accommodate all scenarios in Figure 1 and can indicate whether clustering is appropriate (Figure 1B). If clustering is appropriate, normative models could be used to generate features for clustering. The benefit is that referencing samples to a common normative distribution may yield more interpretable clusters than clustering the data directly. Alternatively, normative modeling might indicate that disease is nested within the healthy range (Figure 1C). We propose one structure that could explain our data that combines these interpretations (Supplemental Figure S5). We also note that we did not find evidence for clearly defined clusters on the basis of symptoms alone (Supplemental Figure S1).

We identified two distinct mechanisms through which participants have high hyperactivity: participants either fit the normative model well, suggesting they are at the extreme of a normal axis of variation, or showed patterns of abnormality that were highly individualized but still meaningfully related to symptoms. These reflect a convergence of different biological mechanisms on the same symptoms. In other words, the extreme clinical phenotype is characterized by mechanistic heterogeneity, which is a key feature of many psychiatric disorders, including ADHD (46). We also showed domain specificity because the degree of abnormality correlated with hyperactivity but not inattention.

It is important to differentiate outliers resulting from clinically meaningful activity from outliers secondary to artifacts. Therefore, it is crucial to demonstrate a relationship between deviations and clinical symptoms or behavior to ensure that deviations from the normative model are driven by clinically relevant abnormalities rather than artifacts. One important source of artifactual variation in functional MRI is head motion (47). It is thus significant that normative modeling provides a way to characterize the deviation statistically, enabling clinically relevant deviations to be distinguished from artifactual deviations based on the individualized pattern of abnormality. Head motion is unlikely to underlie the effects we report because we excluded subjects having a high degree of head motion and repeated the analysis after regressing out motion-related components using a similar approach to Pruim *et al.* (48). All conclusions remained valid, and our subjects that were outliers remained outliers.

We identify three areas for future work. First is to evaluate the stability of the abnormality indices over time. It will be particularly interesting to relate the stability to symptom domains that change with time [e.g., ADHD subtypes (14)]. Second, although we employed a relatively large sample in this study, in the future it will be necessary to handle extremely large data sets.^2^ A limitation for Gaussian process models is a poor computational scaling to large numbers of data points. However, the cost for this data set was acceptable (a few seconds per brain location). For larger data sets, there are many solutions; in preliminary work, we found that an alternative approach [Bayesian polynomial regression (49)] scales well at a small cost to accuracy. There are also many more recent innovations in machine learning for scaling Gaussian process regression to very large data sets (50, 51, 52). Third, it is likely that model accuracy can be improved by modeling spatial correlations using spatial statistics (53).

In conclusion, we demonstrated a normative modeling approach for mapping 1) associations between brain function and behavior and 2) the overall deviation of each subject from the normative model. This approach provides a natural and elegant framework to study clinical conditions in relation to normal functioning without requiring categorical partitioning of the cohort. Instead, disease can be considered as an extremum of the normal range or as—possibly idiosyncratic—deviation from normal functioning. We anticipate that normative modeling will have broad applicability to parsing heterogeneity in many clinical conditions.

## Footnotes

1 We follow the naming convention introduced by the Human Connectome Project, which refers to different brain locations as “brainordinates.” These can reference either vertices on the cortical sheet or subcortical voxels.

2 For example, the UK Biobank sample (www.ukbiobank.ac.uk) aims to scan 100,000 individuals.

Appendix A Supplementary material cited in this article is available online at 10.1016/j.biopsych.2015.12.023.

## References

[1] Kapur S., Phillips A.G., Insel T.R. (2012). Why has it taken so long for biological psychiatry to develop clinical tests and what to do about it?. Mol Psychiatry.

[2] Insel T., Cuthbert B., Garvey M., Heinssen R., Pine D.S., Quinn K. (2010). Research Domain Criteria (RDoC): Toward a new classification framework for research on mental disorders. Am J Psychiatry.

[3] Schumann G., Binder E.B., Holte A., de Kloet E.R., Oedegaard K.J., Robbins T.W. (2014). Stratified medicine for mental disorders. Eur Neuropsychopharmacol.

[4] Cuthbert B.N. (2014). The RDoC framework: Facilitating transition from ICD/DSM to dimensional approaches that integrate neuroscience and psychopathology. World Psychiatry.

[5] Weinberger D.R., Goldberg T.E. (2014). RDoCs redux. World Psychiatry.

[6] Karalunas S.L., Fair D., Musser E.D., Aykes K., Iyer S.P., Nigg J.T. (2014). Subtyping attention-deficit/hyperactivity disorder using temperament dimensions toward biologically based nosologic criteria. JAMA Psychiatry.

[7] Fair D.A., Bathula D., Nikolas M.A., Nigg J.T. (2012). Distinct neuropsychological subgroups in typically developing youth inform heterogeneity in children with ADHD. Proc Natl Acad Sci U S A.

[8] van Hulst B.M., de Zeeuw P., Durston S. (2015). Distinct neuropsychological profiles within ADHD: A latent class analysis of cognitive control, reward sensitivity and timing. Psychol Med.

[9] Costa Dias T.G., Iyer S.P., Carpenter S.D., Cary R.P., Wilson V.B., Mitchell S.H. (2015). Characterizing heterogeneity in children with and without ADHD based on reward system connectivity. Dev Cogn Neurosci.

[10] Lamers F., de Jonge P., Nolen W.A., Smit J.H., Zitman F.G., ATF Beekman (2010). Identifying depressive subtypes in a large cohort study: Results from the Netherlands Study of Depression and Anxiety (NESDA). J Clin Psychiatry.

[11] van Loo H.M., de Jonge P., Romeijn J.-W., Kessler R.C., Schoevers R.A. (2012). Data-driven subtypes of major depressive disorder: A systematic review. BMC Med.

[12] Bell M.D., Corbera S., Johannesen J.K., Fiszdon J.M., Wexler B.E. (2013). Social cognitive impairments and negative symptoms in schizophrenia: Are there subtypes with distinct functional correlates?. Schizophr Bull.

[13] Brodersen K.H., Deserno L., Schlagenhauf F., Lin Z., Penny W.D., Buhmann J.M. (2013). Dissecting psychiatric spectrum disorders by generative embedding. Neuroimage Clin.

[14] Lahey B.B., Pelham W.E., Loney J., Lee S.S., Willcutt E. (2005). Instability of the DSM-IV subtypes of ADHD from preschool through elementary school. Arch Gen Psychiatry.

[15] Mourao-Miranda J., Hardoon D.R., Hahn T., Marquand A.F., Williams S.C.R., Shawe-Taylor J. (2011). Patient classification as an outlier detection problem: An application of the One-Class Support Vector Machine. Neuroimage.

[16] Ziegler G., Ridgway G.R., Dahnke R., Gaser C.., Alzheimer’s Disease Neuroimaging Initiative (2014). Individualized Gaussian process-based prediction and detection of local and global gray matter abnormalities in elderly subjects. Neuroimage.

[17] Rezek I., Beckmann C. (2012). Models of Disease Spectra..

[18] Erus G., Battapady H., Satterthwaite T.D., Hakonarson H., Gur R.E., Davatzikos C. (2015). Imaging patterns of brain development and their relationship to cognition. Cereb Cortex.

[19] Tripp G., Wickens J.R. (2008). Research review: Dopamine transfer deficit: A neurobiological theory of altered reinforcement mechanisms in ADHD. J Child Psychol Psychiatry.

[20] Plichta M.M., Scheres A. (2014). Ventral-striatal responsiveness during reward anticipation in ADHD and its relation to trait impulsivity in the healthy population: A meta-analytic review of the fMRI literature. Neurosci Biobehav Rev.

[21] Reynolds B. (2006). A review of delay-discounting research with humans: Relations to drug use and gambling. Behav Pharmacol.

[22] Ainslie G. (1975). Specious reward—behavioral theory of impulsiveness and impulse control. Psychol Bull.

[23] Kirby K.N. (2009). One-year temporal stability of delay-discount rates. Psychon Bull Rev.

[24] Rasmussen C., Williams C.K.I. (2006). Gaussian Processes for Machine Learning.

[25] Franke K., Ziegler G., Kloeppel S., Gaser C., Alzheimer’s Disease Neuroimaging Initiative (2010). Estimating the age of healthy subjects from T-1-weighted MRI scans using kernel methods: Exploring the influence of various parameters. Neuroimage.

[26] Dosenbach N.U.F., Nardos B., Cohen A.L., Fair D.A., Power J.D., Church J.A. (2010). Prediction of individual brain maturity using fMRI. Science.

[27] Gur R.C., Calkins M.E., Satterthwaite T.D., Ruparel K., Bilker W.B., Moore T.M. (2014). Neurocognitive growth charting in psychosis spectrum youths. JAMA Psychiatry.

[28] Cao B., Mwangi B., Hasan K.M., Selvaraj S., Zeni C.P., Zunta-Soares G.B. (2015). Development and validation of a brain maturation index using longitudinal neuroanatomical scans. Neuroimage.

[29] Marquand A., Howard M., Brammer M., Chu C., Coen S., Mourao-Miranda J. (2010). Quantitative prediction of subjective pain intensity from whole-brain fMRI data using Gaussian processes. Neuroimage.

[30] Hyun J.W., Li Y., Gilmore J.H., Lu Z., Styner M., Zhu H. (2014). SGPP: Spatial Gaussian predictive process models for neuroimaging data. Neuroimage.

[31] Jaynes E. (2003). Probability Theory: The Logic of Science.

[32] Beirlant J., Goegebeur Y., Teugels J., Segers J. (2004). Statistics of Extremes: Theory and Applications.

[33] Coles S. (2001). An Introduction to Statistical Modeling of Extreme Values.

[34] Van Essen D.C., Smith S.M., Barch D.M., Behrens T.E.J., Yacoub E., Ugurbil K. (2013). The WU-Minn Human Connectome Project: An overview. Neuroimage.

[35] Glasser M.F., Sotiropoulos S.N., Wilson J.A., Coalson T.S., Fischl B., Andersson J.L. (2013). The minimal preprocessing pipelines for the Human Connectome Project. Neuroimage.

[36] Ugurbil K, Xu J, Auerbach EJ, Moeller S, Vu AT, Duarte-Carvajalino JM (2013). Pushing spatial and temporal resolution for functional and diffusion MRI in the Human Connectome Project. Neuroimage.

[37] Delgado M.R., Nystrom L.E., Fissell C., Noll D.C., Fiez J.A. (2000). Tracking the hemodynamic responses to reward and punishment in the striatum. J Neurophysiol.

[38] Achenbach T.M. (2009). The Achenbach System of Empirically Based Assessemnt (ASEBA): Development, Findings, Theory, and Applications.

[39] Barch D.M., Burgess G.C., Harms M.P., Petersen S.E., Schlaggar B.L., Corbetta M. (2013). Function in the human connectome: Task-fMRI and individual differences in behavior. Neuroimage.

[40] Myerson J., Green L., Warusawitharana M. (2001). Area under the curve as a measure of discounting. J Exp Anal Behav.

[41] Wesley M.J., Bickel W.K. (2014). Remember the future II: Meta-analyses and functional overlap of working memory and delay discounting. Biol Psychiatry.

[42] Insel TR, Cuthbert BN (2015). Brain disorders? Precisely. Science.

[43] Marquand A.F., Brammer M., Williams S.C.R., Doyle O.M. (2014). Bayesian multi-task learning for decoding multi-subject neuroimaging data. Neuroimage.

[44] Marquand AF, Williams SCR, Doyle OM, Rosa MJ (2014). Full Bayesian multi‐task learning for multi‐output brain decoding and accommodating missing data. In: Proceedings of the 2014 International Workshop on Pattern Recognition in Neuroimaging, Tübingen, Germany. New York: IEEE Press, 1–4.

[45] Wolfers T., Buitelaar J.K., Beckmann C., Franke B., Marquand A.F. (2015). From estimating activation locality to predicting disorder: A review of pattern recognition for neuroimaging-based psychiatric diagnostics. Neurosci Biobehav Rev.

[46] Faraone S., Asherson P., Banaschewski T., Biederman J., Buitelaar J.K., Ramos-Quiroga J. (2015). Attention deficit/hyperactivity disorder. Nat Rev Dis Primers.

[47] Van Dijk K.R.A., Sabuncu M.R., Buckner R.L. (2012). The influence of head motion on intrinsic functional connectivity MRI. Neuroimage.

[48] Pruim R.H.R., Mennes M., van Rooij D., Llera A., Buitelaar J.K., Beckmann C.F. (2015). ICA-AROMA: A robust ICA-based strategy for removing motion artifacts from fMRI data. Neuroimage.

[49] Bishop C. (2006). Pattern Recognition and Machine Learning.

[50] Snelson E, Ghahramani Z (2006): Sparse Gaussian Processes using Pseudo-inputs. Proceedings of the 2005 Conference. In: Weiss Y, Scholkopf B, Platt J, editors. Advances in Neural Information Processing Systems 18. Cambridge, MA: The MIT Press.

[51] Filippone M, Engler R (2015): Enabling scalable stochastic gradient-based inference for Gaussian processes by employing the Unbiased LInear System SolvEr (ULISSE). ArXiv:1501.05427v3 [stat.ME].

[52] Saatci Y. (2011). Scalable Inference for Structured Gaussian Process Models.

[53] Gelfand A., Diggle P., Fuentes M., Guttorp P. (2010). Handbook of Spatial Statistics Boca Raton.

