# Supplementary material for: Understanding Heterogeneity in Clinical Cohorts Using Normative Models: Beyond Case-Control Studies
Source: Biol Psychiatry. 2016 Oct 1;80(7):552–61. doi: 10.1016/j.biopsych.2015.12.023 (PMC5023321; doi:10.1016/j.biopsych.2015.12.023)
Supplement: Supplementary file 1 — Supplementary Material [file mmc1.pdf]

Understanding Heterogeneity in Clinical Cohorts Using Normative Models: Beyond Case Control Studies

Supplemental Information

Supplemental Methods

Overview of Analytical Approach

The analytical procedure we propose is summarized in Figure 2 in the main text and consists of the following five steps: (i) conventional neuroimaging data processing (e.g., motion correction, spatial normalization and smoothing); (ii) estimate regional normative models using Gaussian process (GP) regression. This can be performed on a single cohort using cross-validation or by learning the normative range on a healthy cohort then applying the model to a clinical cohort; (iii) estimate the deviation at each brain region to construct a normative probability map (NPM; 1); (iv) generate a subject level abnormality index by fitting an extreme value distribution to the regional deviations. This can then be correlated with clinical variables; (v) examine deviations more closely by thresholding the NPMs, for example using false discovery rate (FDR) correction to account for multiple comparisons (2). The approach is applicable to most types of neuroimaging data (e.g., structural MRI, fMRI) both at the voxel/vertex level and using regional summary measures. While not our focus in the present work, normative modeling can also be applied to fMRI timeseries, in which case additional steps are necessary (e.g., high-pass filtering and addressing autocorrelation).

Gaussian Process Regression

We provide a brief introduction to GP inference here, the reader is referred to Rasmussen and Williams (3) for a more detailed treatment. Formally, a Gaussian process specifies a distribution over functions, such that any finite number of elements has a joint Gaussian distribution. A GP can be denoted by $\mathcal{N}\left(m(x), k(x, x')\right)$ and is uniquely specified by a mean ($m(x)$) and covariance ($k(x, x')$) function. Gaussian process models can be used for Bayesian nonlinear regression based on a set of

training data $\mathcal{D} = \{\mathbf{x}_i, y_i \in\}_{i=1}^{N}$, where $\mathbf{x}_i$ are D-dimensional vectors of covariates and $y_i \in \mathbb{R}$ are response (or target) variables. Here, covariates were the delay discounting measures for each reward level (i.e., AUC200 and AUC40K; see main text) and responses were the brain activity at each of approximately 90,000 spatial locations, each estimated independently by fitting a classical general linear model (GLM). In the terminology of (4), this approach can be considered an 'encoding' model. We use an unconstrained latent function ($\mathbf{f} = [f_1, \dots, f_N]^T$) to model the relationships between data points, which is assumed to differ from the true response variables by additive noise, i.e., $y_i = f_i + \epsilon_i$ where $\epsilon_i \sim N(0, \sigma_n^2)$. The goal is to estimate this function from the training data in such a way that it allows us to accurately predict a new target y_* from a new data sample $\mathbf{x}_*$. This proceeds by placing a GP prior distribution over the latent function and computing its posterior distribution using Bayes rule:

$$p(\mathbf{f}|D, \boldsymbol{\theta}) = \frac{p(\mathbf{f}|\mathbf{X}, \boldsymbol{\theta})p(\mathbf{y}|\mathbf{f}, \sigma_n)}{p(D|\boldsymbol{\theta})} = \frac{\mathcal{N}(\mathbf{f}|\mathbf{0}, \mathbf{K})\mathcal{N}(\mathbf{y}|\mathbf{f}, \sigma_n^2\mathbf{I})}{\mathcal{N}(\boldsymbol{y}|\mathbf{0}, \mathbf{K} + \sigma_n^2\mathbf{I})} \quad (1)$$

We use $\boldsymbol{\theta}$ to denote any parameters on which the prior distribution depends, $\mathbf{y} = [y_1, \dots, y_N]^T$ to collect all response variables and define $\mathbf{X} = [\mathbf{x}_1, \dots, \mathbf{x}_N]^T$ as a matrix of covariates. We denote the covariance of the Gaussian prior evaluated at the data points by $\mathbf{K}$, which depends on the covariates and the parameters (see below). Under the Gaussian noise model specified above, GP regression has the attractive property that the predictive distribution can be computed exactly, which is achieved by writing the joint Gaussian distribution of the training and test data

$$\begin{bmatrix} \mathbf{y} \\ f_* \end{bmatrix} \sim \mathcal{N}\left(\mathbf{0}, \begin{bmatrix} \mathbf{K} + \sigma_n^2\mathbf{I} & \mathbf{k}_* \\ \mathbf{k}_*^T & k_{**} \end{bmatrix}\right)$$

Where $\mathbf{k}_*$ is a vector containing covariances between the training and test data, f_* denotes the function value evaluated at the test point ($\mathbf{x}_*$), and k_{**} is the variance of the test point.

The predictive distribution for the test point can then be derived using standard identities for partitioned Gaussians:

$$p(f_*|D, \mathbf{x}_*, \boldsymbol{\theta}) \quad = \mathcal{N}(f_*|\hat{f}, \sigma_*^2) \quad (2)$$

$$\hat{f} \quad = \mathbf{k}_*^T(\mathbf{K} + \sigma_n^2\mathbf{I})^{-1}\mathbf{y} \tag{3}$$

$$\sigma_*^2 \quad = k_{**} - \mathbf{k}_*^T(\mathbf{K} + \sigma_n^2\mathbf{I})^{-1}\mathbf{k}_* \tag{4}$$

These are the canonical prediction equations for GP regression and describe the prediction of a noise-free data point. To accommodate noise on the predicted responses (i.e., to compute $\hat{y}$), we simply take the predictive mean given by (3) and add the predictive variance derived from the training set (σ_n^2) to (4).

The ability to flexibly specify many different forms for the covariance is the key to the modeling flexibility of the GP framework. While many forms for the covariance function are possible, in this work, we use a covariance function that combines linear and non-linear terms, i.e.,:

$$k(\mathbf{x}_i, \mathbf{x}_j) = \mathbf{x}_i^T\mathbf{x}_j + \sigma_f \exp\left(-\frac{1}{2}(\mathbf{x}_i - \mathbf{x}_j)^T\mathbf{\Lambda}(\mathbf{x}_i - \mathbf{x}_j)\right)$$

Where σ_f is an amplitude parameter for the nonlinear component and $\mathbf{\Lambda}$ is a diagonal matrix with ℓ_d^{-2} along the leading diagonal. These are 'automatic relevance determination' parameters (5) that encode a different covariance length scale for each dimension. Each of these encodes how smoothly the interpolating function varies in covariate space along each dimension. In practice, these parameters can be used to down-weight irrelevant dimensions in the input space (by setting the length scale to a very high value) or emphasize important dimensions (low value). For example, if only the delay discounting scores from the high reward condition are useful for predicting brain activity, it is advantageous to down-weight all other covariates. The length scale parameters are themselves potentially valuable outcomes from the model, particularly in cases where many covariates are used to predict the response, because they directly show the informative content of each covariate. Training a GP model refers to finding the optimal values for the model parameters ($\boldsymbol{\theta}$) which are: $\ell_1, \ldots, \ell_D, \sigma_n$ and σ_f. This is conveniently and efficiently achieved by maximizing the logarithm of the model evidence ($p(D|\boldsymbol{\theta})$, the denominator of (1)) which encodes the optimal trade-off between data fit and model complexity under the assumptions of the model.

Deriving a Subject-Level Abnormality Index Using Extreme Value Statistics

Extreme value statistics are concerned with modeling the behavior of random variables in the tail of their distribution. In other words, they are used for the prediction of extreme deviations from a distribution mean, which are by definition rare events. Extreme value statistics can be used to determine the probability of events more extreme than any that have been observed from an existing sample and have been applied, for example, to predicting unusually large floods or stock market crashes (6, 7). There are two main approaches to modeling extreme deviations: (i) 'block maxima' approaches, which involve summarizing the data by the maximum over each block (e.g., an annual maximum) and (ii) 'peaks over threshold' which involves summarizing the data by taking all values that exceed a fixed threshold. The block maxima approach is the most appropriate for our purposes; we consider each subject as a 'block' which we summarize by taking a robust mean of his or her maximum 1% , minimum 1% or maximum 1% absolute value of normative probability scores ('positive-', 'negative-' or 'absolute deviations', respectively). To make probabilistic subject-level inferences about these deviations, they can be fit to a generalized extreme value (GEV) distribution. The GEV distribution is the most appropriate choice for these purposes because of an important theoretical result that states that – regardless of the data generating distribution – the maximum of a sample of any independent random variables can only asymptotically converge to one of three distributions (a Gumbel, Weibull or Fréchet distribution). This is known as the 'extreme value theorem' (8) and plays a similar role in extreme value statistics to the central limit theorem for describing the asymptotic behavior of a mean of a sample of random variables. All of these limiting distributions are subsumed by the GEV distribution.

Implementation

All methods were implemented in MATLAB using custom scripts that make use of functionality provided by the Gaussian processes for machine learning (GPML) toolbox (www.gaussianprocess.org/gpml/code/). We employ a conjugate gradient optimizer (minimize.m,

part of the GPML toolbox) to optimize model parameters and optimize all parameters in the log domain to ensure positivity. We refer the reader elsewhere for full details (3). We fit GEV distributions using maximum likelihood using a custom MATLAB implementation.

Data Sample

For this work we use neuroimaging, clinical and behavioral data from the '500 subjects' release of the Human Connectome Project (HCP; www.humanconnectome.org). The HCP aims to acquire exceptionally high-quality neuroimaging (including structural MRI, resting state fMRI and task-based fMRI), behavioral, clinical and genetic data from 1200 healthy individuals. We refer the reader to prior publications for full details surrounding the motivation (9), behavioral data and task design (10), data acquisition (11) and preprocessing (12). Briefly however, all participants included in this report (N = 491, aged 22-35, 288 females) were born in Missouri to families that included twins and at least one other sibling. Specifically, the sample analyzed here included data from 215 families, containing 240 twins, of whom 117 were monozygotic. Note that for some twin pairs only one was included in the present sample (see ref 9 for further details). All subjects had no known history of psychiatric, neurological or medical disorder known to influence brain function and completed a battery of psychometric tests in a range of cognitive, affective, personality, somatosensory and physical domains (see 10). The demographic and clinical characteristics of the Human Connectome Project sample used in this work are provided in Table 1 in the main text.

All participants completed a revised version[1] of the semi-structured assessment for the genetics of alcoholism (SSAGA-IV; 13), a diagnostic instrument that is based on DSM-IV criteria, is well-validated and has been used in many large scale studies (e.g., 14). The SSAGA assesses many diagnostic categories including adult ADHD, substance use, mood, anxiety and eating disorders. We used the SSAGA/DSM-IV to screen participants. Participants also completed the Achenbach Adult Self-Report instrument (ASR; 15) which we used to measure clinical symptoms on the basis of DSM-

[1] See https://niaaagenetics.org/coga_instruments/resources.html

IV criteria. Here, we used the ASR scales for inattention and impulsivity/hyperactivity as indicators of ADHD symptoms.

Measures of Trait Impulsivity Derived From Delay Discounting

All participants performed a well-validated delay discounting task (10) that aimed to identify the extent to which future rewards are devalued relative to immediate rewards and to provide an index of trait impulsivity. An 'adjusting amount' variant (16) was used in which delays are fixed and reward amounts are adjusted on a trial-by-trial basis in order to rapidly identify an indifference point, where participants are equally likely to choose an smaller immediate reward over a larger delayed reward. Two delayed reward magnitudes were used, a larger reward ($40,000) and a smaller reward ($200) and the area under the curve (AUC) approach was used to summarize all delays evaluated (17), providing a simple and model-free overall index of how steeply each participant discounts reward over time for each reward magnitude ('AUC40K' and 'AUC200').[2]

Functional MRI Task

We used data from the HCP incentive processing (i.e., gambling) task as a biological indicator of reward processing. Similar tasks have been used in many prior reports (e.g., 18, 19) and the one used here required participants to play a card game where they were asked to guess the number on a mystery card in order to win or lose money. Subjects were informed that cards were numbered from 1-9 and were asked to indicate whether the mystery card was greater than or less than 5 by pressing one of two buttons on a response box. Feedback was provided for each trial according to whether the subject won (a green up arrow with $1) or lost (a red down arrow, with -$0.50) money or whether the trial was neutral (a gray double headed arrow with the number 5). Two fMRI runs were acquired with trials arranged in blocks of 8 trials (2 mostly win blocks, 2 mostly lose blocks interleaved with 4 15-s fixation blocks). See (10) for full details.

[2] See http://www.humanconnectome.org/documentation/S500/ for a detailed description of the delay discounting task

Functional MRI Data Acquisition and Processing

Whole-brain echo-planar imaging (EPI) data were acquired on a modified 3T Siemans Skyra MRI system (www.siemens.com) according to the standard HCP multiband fMRI acquisition protocols. Full details are provided in Ugurbil *et al.* (11), but sequence parameters were TR = 720 ms, TE = 33.1 ms, flip angle = 52°, BW = 2290 Hz/Px, in-plane FOV = 208 × 180 mm, 72 slices, 2.0 mm isotropic voxels. Data were processed according to the HCP surface-based pipelines (12) that involved gradient unwarping, motion correction, fieldmap-based EPI distortion correction, boundary-based registration of EPI to structural T1-weighted scan, non-linear registration into MNI152 space, and grand-mean intensity normalization. The data were projected onto the cortical sheet from each cerebral hemisphere then both hemispheres and volumetrically processed subcortical structures were combined into a single data format. Finally, fMRI data were smoothed with an 8 mm kernel that respected the geometry of the brain, implemented by a geodesic surface smoothing algorithm for cortical structures and a parcel-constrained volumetric smoothing algorithm for subcortical structures (see 12 for full details).

A GLM was then estimated using the FSL software package version 5.06 (http://fsl.fmrib.ox.ac.uk/). Two regressors were constructed from the win and loss blocks which were then convolved with a canonical double-gamma haemodynamic response function and combined with the temporal derivatives of each main regressor. These were treated as nuisance regressors and served to accommodate slight variations in slice timing or in the haemodynamic response. Data were pre-whitened using a version of FSL-FILM customized to accommodate surface data, the model and data were high-pass filtered (200 s cutoff) then fixed-effects GLMs were estimated using FSL-FLAME: first for each run, then to combine both runs into a single model for each participant. Finally, the contrast maps between rewarded blocks and baseline for each subject were used as response variables for the normative model.

Cross Validation and Performance Assessment

To ensure unbiased estimates of accuracy and the participant-level deviation from the normative model, model estimation was embedded within a 10-fold cross-validation procedure that preserved the family structure within the sample. This is important because the sample includes twins and their siblings, which would bias prediction accuracy and the normative probability maps if not accounted for (see *Data Sample* above for details about the family structure). To account for this structure, we ensured that for each fold, all subjects from each family were in either the training or test partitions, but were not split across both. The performance of the classifier on unseen data samples was assessed using the mean squared error (MSE), which was computed as:

$$MSE = \frac{1}{N}\sum_{i=1}^{N}(y_i - \widehat{y}_i)^2$$

A problem with using the MSE directly is that its precise value depends on the scale of the data, which complicates the comparison of different MSE scores across different brain ordinates. We therefore follow Rasmussen and Williams (3) and standardize this quantity by dividing by the variance in the response variables, yielding the standardized MSE.

Supplementary Results

Clinical Measures

The relationship between ASR scores for inattention and hyperactivity are shown in Figure S1. Both symptom domains are highly correlated and reveal little structure to distinguish subgroups of participants.

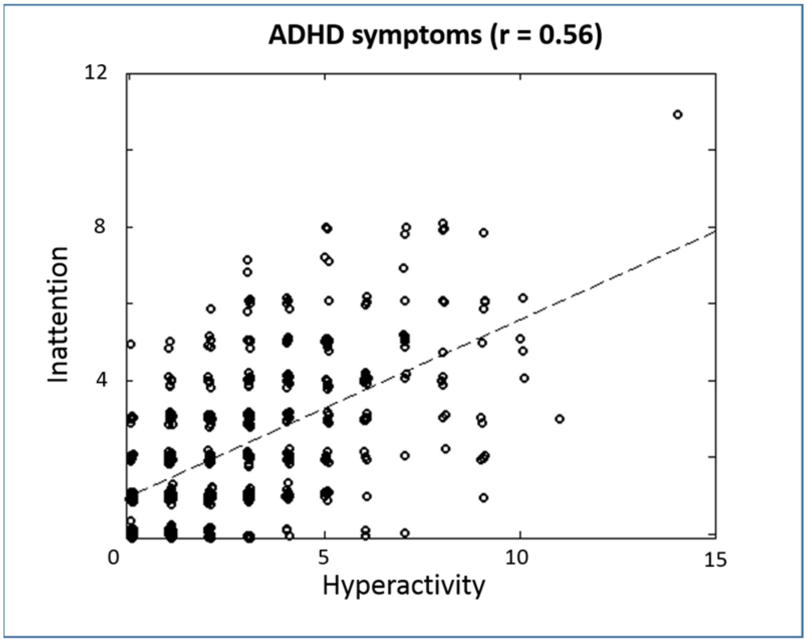

Figure S1. Scatterplot showing Achenbach Self Report symptom scores for hyperactivity inattention. A small amount of jitter has been added to show the approximate density of points at each location.

Brain Regions Engaged by the Normative Model

As is shown in Figure 4 in the main text, the normative model predicts that increased delay discounting is associated with increased activity in lateral prefrontal, superior medial prefrontal, middle temporal, parietal and lateral visual cortices (especially in the right hemisphere) in addition to dorsal parts of caudate nuclei, cerebellum and brainstem regions. Increased delay discounting is also associated with decreased activity in medial occipital cortex, bilateral thalamus, left pallidum and right hippocampus.

Determining the Clinical Correlates of the Subject Level Abnormality Index

The relationship between the absolute deviance from the normative model and self-reported inattention scores are shown in Figure S2. Comparison with Figure 5 in the main text shows that the subjects having the greatest deviation from the normative model did not have high hyperactivity symptoms. This, taken together with the observation that deviation from the normative model did not correlate with inattention symptoms (see main text), leads us to conclude that deviations from the normative model were less strongly related to inattention than hyperactivity.

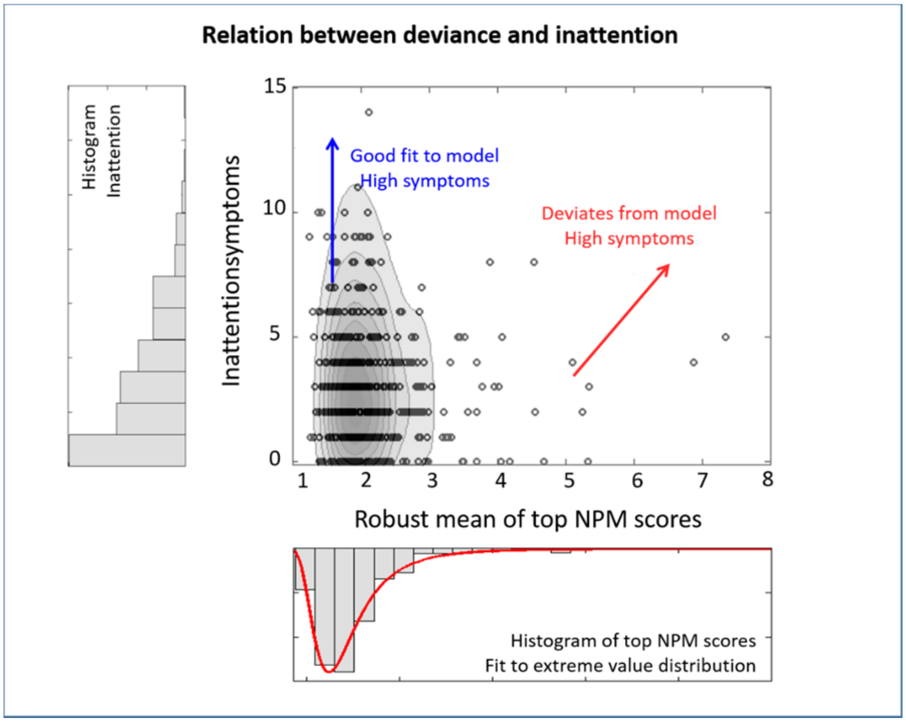

Figure S2. The relationship between the overall deviance from the normative model and self-reported inattention scores (center), along with the histograms of the component measures (left and bottom). This figure allows us to determine whether subjects that have high clinical symptoms show a good or a poor fit to the normative model. For illustrative purposes, contour lines show the density of points in the figure. Most points fit the normative model well but some of these subjects also score highly on inattention (blue arrow). Subjects who do not fit the normative model generally do not have high inattention symptoms. NPM, normative probability map.

Overlap of Deviations Across Subjects

Together with Figure 6 in the main text, Figure S3 shows the most extreme outlying subjects, ranked by hyperactivity symptoms. Figure S4 shows the overlap between the NPMs for all subjects with activity different from the normative model ($p < 0.05$, FDR corrected). The overlap between subjects was relatively low and there were no brain areas which showed abnormal activity in more than three subjects. Thus the patterns of abnormality were highly individualized.

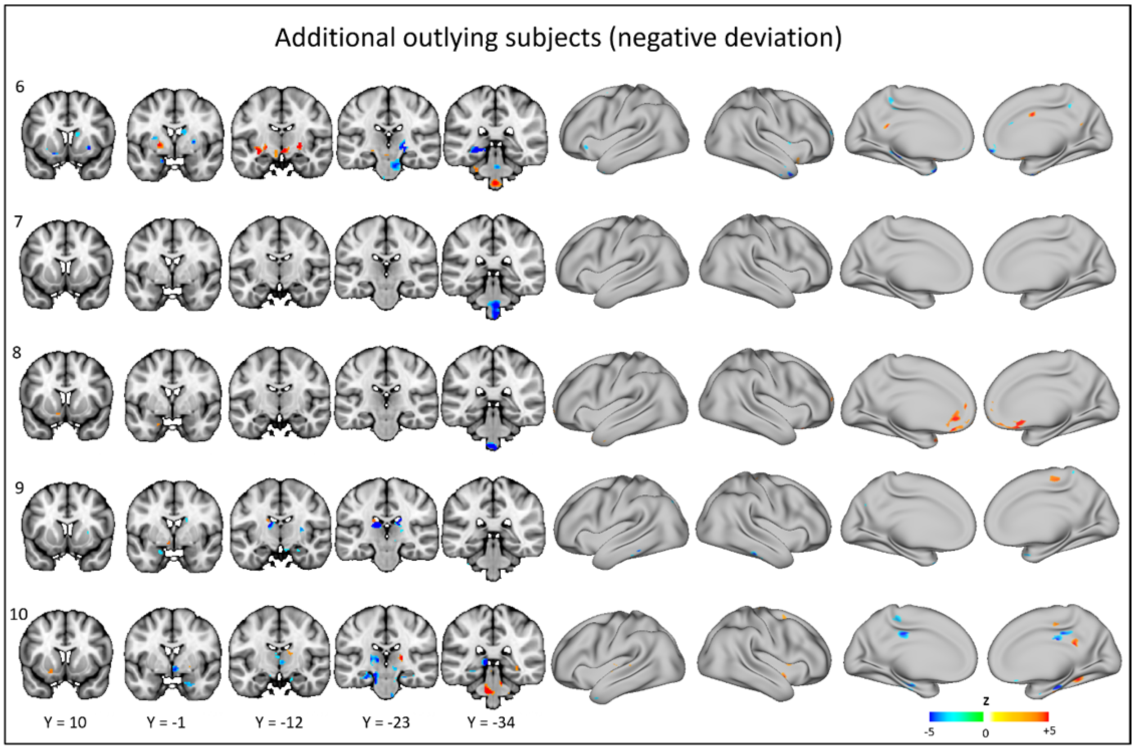

Figure S3. Normative probability maps that describe the brain regions that deviate from the normative model in subjects having the most extreme deviations ($p < 0.05$, false discovery rate corrected). These subjects supplement those presented in Figure 6 in the main text. Warm colors indicate greater activity than would be predicted by the normative model and cool colors indicate reduced activity relative to the normative model. Subjects are ranked by hyperactivity symptom scores with the rank indicated by the small numerals.

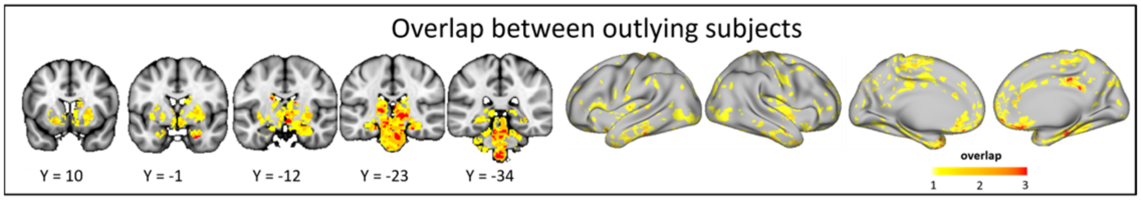

Figure S4. The overlap across subjects of all brain regions that differ from the normative model according to the NPM Z scores ($p < 0.05$, false discovery rate corrected).

Interpretation of Deviations

Figure S5 shows one possible interpretation of the deviations plotted in Figure 5 in the main text. See main text for a description.

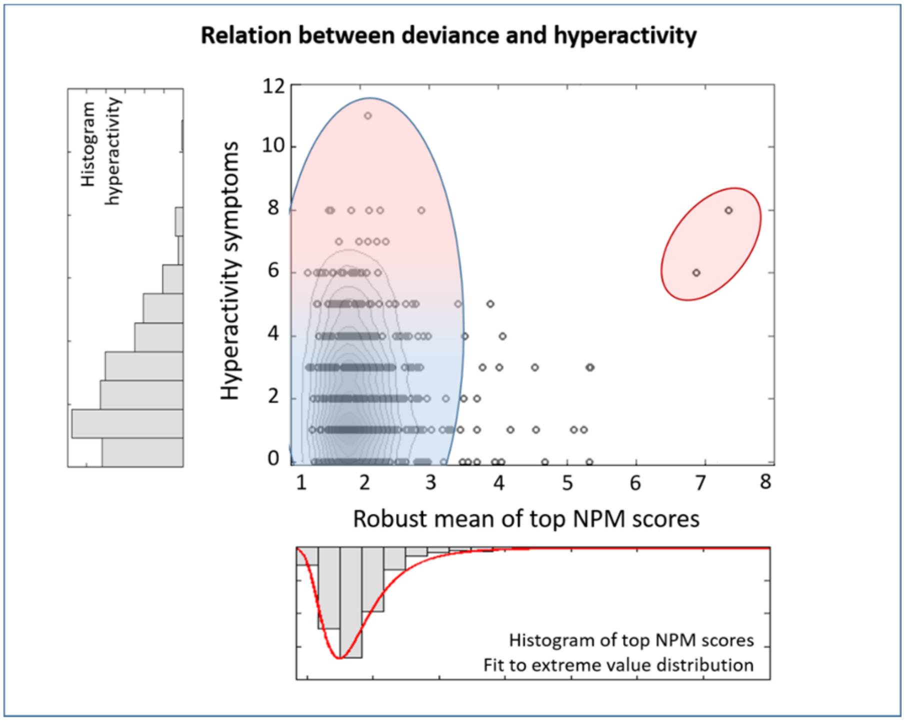

Figure S5. One possible interpretation of the pattern of deviations observed in the dataset examined here. Symptoms may arise through mechanisms that are well-captured by the normative model (large ellipse), or by idiosyncratic deviations from the normative model (small ellipse). See main text for details.

Supplemental References

1. Ziegler G, Ridgway GR, Dahnke R, Gaser C, Alzheimer's Dis N (2014): Individualized Gaussian process-based prediction and detection of local and global gray matter abnormalities in elderly subjects. *Neuroimage*. 97:333-348.
2. Genovese CR, Lazar NA, Nichols T (2002): Thresholding of statistical maps in functional neuroimaging using the false discovery rate. *Neuroimage*. 15:870-878.
3. Rasmussen CE, Williams C (2006): *Gaussian Processes for Machine Learning*. MIT Press.
4. Naselaris T, Kay KN, Nishimoto S, Gallant JL (2011): Encoding and decoding in fMRI. *NeuroImage*. 56:400-410.
5. Neal R (1996): Probabilistic inference using Markov-chain Monte Carlo methods.
6. Beirlant J, Goegebeur Y, Teugels J, Segers J (2004): *Statistics of Extremes: Theory and Applications*. Sussex, England: John Wiley and Sons.
7. Coles S (2001): *An Introduction to Statistical Modeling of Extreme Values*. Springer.
8. Gnedenko BV, Kolmogorov AV (1954): *Limit Distributions for Sums of Independent Random Variables*. Addison Wesley.
9. Van Essen DC, Smith SM, Barch DM, Behrens TEJ, Yacoub E, Ugurbil K, et al. (2013): The WU-Minn Human Connectome Project: An overview. *Neuroimage*. 80:62-79.
10. Barch DM, Burgess GC, Harms MP, Petersen SE, Schlaggar BL, Corbetta M, et al. (2013): Function in the human connectome: Task-fMRI and individual differences in behavior. *Neuroimage*. 80:169-189.
11. Ugurbil K, Xu J, Auerbach EJ, Moeller S, Vu AT, Duarte-Carvajalino JM, et al. (2013): Pushing spatial and temporal resolution for functional and diffusion MRI in the Human Connectome Project. *Neuroimage*. 80:80-104.
12. Glasser MF, Sotiropoulos SN, Wilson JA, Coalson TS, Fischl B, Andersson JL, et al. (2013): The minimal preprocessing pipelines for the Human Connectome Project. *Neuroimage*. 80:105-124.
13. Bucholz KK, Cadoret R, Cloninger CR, Dinwiddie SH, Hesselbrock VM, Nurnberger JI, et al. (1994): A new, semistructured psychiatric interview for use in genetic-linkage studies - a report on the reliability of the SSAGA. *Journal of Studies on Alcohol*. 55:149-158.
14. Hesselbrock M, Easton C, Bucholz KK, Schuckit M, Hesselbrock V (1999): A validity study of the SSAGA - a comparison with the SCAN. *Addiction*. 94:1361-1370.
15. Achenbach TM (2009): *The Achenbach System of Empirically Based Assessemnt (ASEBA): Development, Findings, Theory, and Applications*. Burlington, VT: University of Vermont Research Center for Children, Youth and Families.
16. Estle SJ, Green L, Myerson J, Holt DD (2006): Differential effects of amount on temporal and probability discounting of gains and losses. *Memory & Cognition*. 34:914-928.
17. Myerson J, Green L, Warusawitharana M (2001): Area under the curve as a measure of discounting. *Journal of the Experimental Analysis of Behavior*. 76:235-243.
18. Delgado MR, Nystrom LE, Fissell C, Noll DC, Fiez JA (2000): Tracking the hemodynamic responses to reward and punishment in the striatum. *Journal of Neurophysiology*. 84:3072-3077.

19. Hariri AR, Brown SM, Williamson DE, Flory JD, de Wit H, Manuck SB (2006): Preference for immediate over delayed rewards is associated with magnitude of ventral striatal activity. *Journal of Neuroscience*. 26:13213-13217.
